# Epidemiology of visceral leishmaniasis among children in Gadarif hospital, eastern Sudan

**DOI:** 10.1186/s12889-016-3875-2

**Published:** 2016-12-07

**Authors:** Mohammed Ahmed A. Ahmed, Ahmed A. Ahmed, Saeed M. Omar, Gamal K. Adam, Tajeldin M. Abdallah, AbdelAziem A. Ali

**Affiliations:** 1 Faculty of Medicine, Gadarif University, Gadarif, Sudan; 2 Department of Obstetrics and Gynecology, Faculty of Medicine, Kassala University, P.O. Box 496, Kassala, Sudan

**Keywords:** Visceral leishmaniasis, Epidemiology, Children, Anaemia, Sudan

## Abstract

**Background:**

Since 1900s, visceral leishmaniasis (VL) has been among the most important health problems in Sudan, particularly in the endemic areas such as eastern and central regions.

**Methods:**

This was a cross sectional, hospital-based study conducted from 1^st^ January 2015 to 31^st^ December 2015 to investigate the epidemiological factors of VL in Gadarif hospital, eastern Sudan.

**Results:**

During the study period there were 47 identified children with VL among 145 suspected cases. The most common clinical presentations were fever (47, 100%), pallor (47, 100%), weight loss (40, 85.1%), splenomegaly (37, 78.7%), lymphadenopathy (33, 70.2%), vomiting (32, 68%) cough (28, 59%), loss of appetite (22, 46.8%), diarrhoea (17, 36.1%) and jaundice (5, 10.6%). With regard to the outcome after short term follow up 37 patients (78.8%) improved without complications, while 3 (6.4%, 2 (4.3%), 2 (4.3%), 1 (2.1%), 1 (2.1%) and 1 (2.1%) developed pneumonia, otitis media, septicaemia, urinary tract infection, parasitic infestation and PKDL respectively. Lower mean of haemoglobin level was observed among the VL cases in comparison with the suspected cases (in whom VL was excluded) haemoglobin level {8.9 (3.1) Vs 11 (6.3), *P* = 0.021}. Again more proportion of anaemic (47 (100%) Vs 14 (14.2%), *P* = 0.000) and severely anaemic (23 (48.9%) Vs 2 (2%), *P* = 0.006) patients was detected among the infected children. Using logistic regression analyses there was significant association between rural residence *(CI = 1.5–24, OR = 19.1, P =* 0.023*)*, male gender *(CI = 6.6–18.7, OR = 6.4, P =* 0.001*)* and VL among children.

**Conclusions:**

While there is an advance in prevention and management of visceral leishmaniasis our results indicate that VL is still a public health problem with its severe complications among children in eastern Sudan.

## Background

Visceral leishmaniasis (VL) or kala-azar is a parasitic infection caused by two leishmanial species, *L. donovani* or *L. infantum/ chagasi*. The infection is transmitted by the bite of infected sand fly *Phlebotomus argentipes. L. infantum/ chagasi* infects mostly children and immune-suppressed individuals, whereas *L. donovani* infects all age groups [1]. Anaemia, weight loss, hepatosplenomegaly, lymphadenopathy, prolonged fever, anorexia, loss of appetite, pancytopenia and hair changes are the most common clinical presentation among the children [2]. Bacterial super-infection such as pneumonia, septicemia, otitis media, urinary tract infections and skin infections are the major complications leading to death in children with VL [3]. Other complications among children include parasitic infestations of the alimentary tract and post-kala-azar dermal leishmaniasis (PKDL) [1]. Worldwide the annual incidence and prevalence of kala-azar cases are 0.5 and 2.5 million, respectively [1]. According to recent reports, leishmaniasis is endemic in 98 countries, and around 1.3 million new cases are reported every year, with an estimated 20,000 to 40,000 deaths every year [4]. The vast majority (more than 90%) of cases are in Sudan, India, Bangladesh, Nepal and Brazil. Children constitute 7-10% of such cases in the endemic regions [5]. Since 1900s, visceral leishmaniasis (VL) has been among the most important health problems in Sudan, particularly in the endemic areas such as eastern and central regions [6]. Thus, the current study was conducted to investigate the epidemiological factors of VL among children in Gadarif, eastern Sudan and the results of this study is expected to provide the health planners with fundamental data necessary for the implementation of preventive measures in this area of the country.

## Methods

### Study design and data collection

This was a cross sectional, hospital-based study conducted from 1^st^ January 2015 to 31^st^ December 2015 to investigate the epidemiological factors of VL in Gadarif hospital, eastern Sudan.

The cases included all children ≤14 year old with clinical symptoms of VL and in whom the diagnosis of VL was confirmed by laboratory test. A structured questionnaire was used to gather the socio-demographic characteristics (age, residence, the education and occupation of both parents), duration of the illness, reason of clinical presentation (fever, fatigue, weakness, loss of appetite and weight loss), clinical sign (pallor, jaundice, epistaxis or sign of bleeding tendency, mucocutanous lesion, PKDL, enlarged lymph nodes, spleen and liver), super-infection (pneumonia, otitis media, septicaemia, worm infestation) and patient outcome (improvement, death). Proper systemic examination was performed to each patient by a paediatrician including cardiovascular system, respiratory system, abdomen, musculoskeletal system and central nervous system. Basic tests were performed for every patient on admission and repeated when clinically indicated. These included complete blood count, urine analysis, blood film for malaria, stool analysis and abdominal ultrasound. We looked for the parasite in bone marrow aspirate and other tissues using Giemsa-stain. The diagnosis was confirmed by the visualization of the amastigote form of the parasite by microscopic examination of aspirates from lymph nodes or bone marrow. Specific anti-leishmanial drugs (sodium stibogluconate was the first line while in cases with severe side effects liposomal amphotericine B was the second option) and the aggressive management of any concomitant bacterial or parasitic infections, anaemia, hypovolemia (decreased blood volume) and malnutrition was the treatment for the patients. All patients were under multidisciplinary care and were closely followed during their hospital stay and then every month in the refer clinic.

### Statistics

Data was entered into a computer database and SPSS software (SPSS Inc., Chicago, IL, USA, version 16.0) and double checked before analysis. Analysis of variance was used to compare means and x^2^ was used for categorical variables. Univariate and multivariate analyses were performed. Visceral leishmaniasis was the dependent variable and socio-demographic characteristics were independent variables. Confidence intervals of 95% were calculated and *P* < 0.05 was considered significant.

## Results

During the study period there were 47 identified children with VL among 145 suspected cases. All the studied patients were residents of Gadarif State, their age ranged between 0 and 12 year, and the vast majority of the infected children were of rural residence (28/47, 59.6%). The age distribution of the studied cases was as follows: infants (0 to 2 years) constituted 7 (14.9%) cases; toddlers (3 to 5 years) 17 (36.2%) cases; children (6 to 9 years) 16 (34%) cases and ten- to twelve-year-old constituted 7 (14.9%) of the cases. Out of the total 34 (72.3%) were male and 13 (27.7%) were female giving 2.6:1 male: female ratio. The most common clinical presentations were fever (47, 100%), pallor (47, 100%), weight loss (40, 85.1%), splenomegaly (37, 78.7%), lymphadenopathy (33, 70.2%), vomiting (32, 68%) cough (28, 59%), loss of appetite (22, 46.8%), diarrhoea (17, 36.1%) and jaundice (5, 10.6%). A positive family history for leishmaniasis was seen in (9) 19.1% of the cases. Anaemia (haemoglobin level < 12 g/dl) was detected in all (100%) patients and severe anaemia (haemoglobin level < 7 g/dl) was reported among 23 (48.9%) cases. Severe anaemia was observed in 4, 5, 8 and 6 cases of infant, toddlers, children and ten to twelve year old groups respectively. Interestingly 25.5% (12/47) were initially misdiagnosed and mistreated as cases of febrile illness such as malaria and other. Most of the patients 41 (87.2%) responded to sodium stibogluconate, in 6 patients (12.8%) sodium stibogluconate was stopped due to toxic effects. These patients received libosomal amphotericin B and showed good response. With regard to the outcome after short term follow up 37 patients (78.8%) improved without complications, while 3 (6.4%), 2 (4.3%), 2 (4.3%), 1 (2.1%), 1 (2.1%) and 1 (2.1%) developed pneumonia, otitis media, septicaemia, urinary tract infection, parasitic infestation and PKDL respectively. Lower mean of haemoglobin level was observed among the VL cases in comparison with the suspected cases (in whom VL was excluded), (Table 1). Again more proportion of anaemic (47 (100%) Vs 14 (14.2%), *P* = 0.000) and severely anaemic (23 (48.9%) Vs 2 (2%), *P* = 0.006) patients was detected among the infected children, (Table 1). Using logistic regression analyses there was significant association between rural residence *(CI = 1.5–24, OR = 19.1, P =* 0.023*)*, male gender *(CI = 6.6–18.7, OR = 6.4, P =* 0.001*)* and VL among children, (Table 2).

**Table 1:** Comparison between children with VL and those without VL in Gadarif hospital, Eastern Sudan

Variables	Confirmed cases (*N* = 47)	Not confirmed cases (*N* = 98)	*P*
Age, years	5.1 (3.3)	4.8 (3.1)	0.313
Weight	10.6 (5)	10.6 (4.9)	0.529
Hb, gm\dl	8.9 (3.1)	11 (6.3)	0.021
Rural residence	28 (59.6)	21 (21.4)	0.000
Gender. male	34 (72.3)	29 (29.6)	0.002
Family history, yes	9 (19.1)	24 (24.5)	0.310
Anaemia , yes	47 (100)	14 (14.2)	0.000
Severe anaemia, yes	23 (48.9)	2 (2)	0.006

Data of age, weight and Hb are shown as mean (SD) while data of other variables are shown as number (%) as applicable

**Table 2:** Factors associated with VL among children in Gadarif hospital, eastern Sudan, using univariate and multivariate analyses

Variable	Univariate analyses			Multivariate analyses		
	OR	95% CI	*P*-value	OR	95% CI	*P*-value
Age, year	0.9	0.8–1.0	0.167	0.9	0.4–1.9	0.957
Residence, rural	5.0	2.5–11.5	0.000	19.1	1.5–24	0. 023
Weight, Kg	0.6	1.0–2.0	0.409	0.9	0.8–1.0	0.081
Gender, male	6.2	2.8–13.4	0.000	6.4	6.6–18.7	0.001
Family history, yes	0.7	0.3–1.7	0.474	0.8	0.8–1.9	0.677
Anaemia, yes	45	14–146	0.000	47	3–60.4	0.003

*Abbreviations*: *OR* odds ratio, *CI* confidence interval

## Discussion

This is the first published data on VL among children in Gadarif, eastern Sudan which is populated by 1,7 million inhabitants. The current study showed significant association between residence, male gender and VL among children and nearly one half (48.9%) of VL patients had severe anaemia.. Severe VL epidemics are reported in poor communities and poor people in developing and middle income countries such as Nepal and India [7]. Anaemia is a common public health problem in eastern Sudan where 36.2% of the adults had anaemia [8]; the common causes of anaemia in this area include: chronic illness, followed by nutritional and repeated malaria infection [9]. Severe VL epidemics have been reported in the past: in Southern Sudan, in a context of civil war and at that time VL killed an estimated 100,000 people out of a population of 280,000 between 1984 and 1994 [10]. Rapid urbanization, and human migration are known risk factors that potentiate the spread of the disease. Gadarif state is bordering two neighbouring countries (Ethiopia and Eretria) and characterized by poverty with nomads and refugees and this might explain the prevalence of the infection in this area of Sudan. Interestingly 25.8% of the patients were initially misdiagnosed and mistreated as cases of febrile illness such as malaria. This is attributed to the lack of proper diagnostic facilities and low priority of leishmaniasis in our setting. Furthermore this may be another problem added to the probability of relapses due to loss of follow up and clinical complications. Thus health care providers generally need high clinical suspicion to diagnose VL. The clinical presentation of VL in children in the majority of the cases is more severe than in adults, commonly with severe anaemia. In our cases, fever and pallor, weight loss and lymphadenopathy were the most common clinical presentations. The clinical presentation of VL is similar in the various endemic areas. However lymphadenopathy is rarely found in Indian VL patients but it is frequent in Sudanese VL patients [11]. In a study conducted by *Rai* et al., majority of the patients (98%) presented with fever followed by abdominal distension (47%), pallor (44%), weight loss (43%), diarrhoea (17%), vomiting (15%) and hepatosplenomegaly (83%) [12]. Again and in consistent with our results, weight loss, hepatosplenomegaly, lymphadenopathy and prolonged fever were the most common clinical signs reported by *Walyeldin* and his colleagues in Omdurman Emergency Hospital for Children (OEHC), Sudan, 2006–2008 [6]. The severity of anaemia in children with VL in this study is also seen in other studies [13]. *Alvar J* et al. also mentioned that children with VL suffer more severe anaemia than adults [14]. In our study anaemia was seen in all patients while severe anaemia was reported in 23 cases which is in line with studies conducted in Kashmir and Peshawar where anaemia was seen in all (100%) cases of VL [15, 16]. The reported complications of VL among our investigated children in this study include: pneumonia, otitis media, septicaemia, urinary tract infection, parasitic infestation and PKDL. While PKDL is not rare in Sudanese VL patients, liver impairment and jaundice are a rare finding [13]. Complications in eyes and mucous membranes were seen but rare. The interval between treated VL and PKDL is 0–6 months in Sudan and 6 months to 3 years in India [17]. In this study 85% respond to sodium stibogluconate (Pentostam ®) which is not different from studies in other areas, for example studies conducted by *Kirk* and *Satti* early in 1940 [18] and *Khalil* et al. in 1998 [19]. In agreement with our study *Ali Shah* et al. in Peshawar [16] and a study from Muzaffarabad [20] showed a male to female ratio of 2:1. However in contrast to our study *Qasmi* et al., in Morocco, reported a higher mean age and a female predominance in children with leishmaniasis [20]. Male predominance in our study could be easily justified by the fact that the male children usually work with their fathers in the farms and thus there is greater chance to be exposed to the sand fly. In our study there was significant correlation between VL and rural residence. Visceral leishmaniasis affects poor communities, generally in remote rural areas. Patients and families affected by VL become poorer because of the high direct costs (for example, the costs of VL diagnosis and treatment) and indirect costs (for example, loss of household income) of the disease [7, 21].

## Conclusions

While there is an advance in prevention and management of visceral leishmaniasis our results indicate that VL is still a public health problem with its severe complications among children in eastern Sudan. High clinical suspicion and introducing the preventive measures (prevention of transmission through vector control and community awareness) are needed to reduce the spread of the infection.
